# Properties of Fluorescent Far-Red Anti-TNF Nanobodies

**DOI:** 10.3390/antib7040043

**Published:** 2018-12-15

**Authors:** Ekaterina N. Gorshkova, Grigory A. Efimov, Ksenia D. Ermakova, Ekaterina A. Vasilenko, Diana V. Yuzhakova, Marina V. Shirmanova, Vladislav V. Mokhonov, Sergei V. Tillib, Sergei A. Nedospasov, Irina V. Astrakhantseva

**Affiliations:** 1 Center of Molecular Biology and Biomedicine, Institute of Biology and Biomedicine, Lobachevsky State University, Nizhniy Novgorod 603950, Russia; tworogowa.kseniya@yandex.ru (K.D.E.); kat802@rambler.ru (E.A.V.); vlad.mokhonov@gmail.com (V.V.M.); sergei.nedospasov@gmail.com (S.A.N.); 2 Laboratory of Transplantation Immunology, National Research Center for Hematology, Moscow 125167, Russia; gefimova@gmail.com; 3 Institute of Biomedical Technologies, Nizhny Novgorod State Medical Academy, Nizhniy Novgorod 603005, Russia; yuzhakova-diana@mail.ru (D.V.Y.); shirmanovam@mail.ru (M.V.S.); 4 Engelhardt Institute of Molecular Biology, Russian Academy of Sciences, Moscow 119991, Russia; 5 Lomonosov Moscow State University, Moscow 119991, Russia; sergei.tillib@gmail.com; 6 Institute of Gene Biology, Russian Academy of Sciences, Moscow 119334, Russia

**Keywords:** TNF, fluorescent, nanobodies, sensor, anti-cytokine therapy, autoimmune disease

## Abstract

Upregulation of the expression of tumor necrosis factor (TNF-α, TNF) has a significant role in the development of autoimmune diseases. The fluorescent antibodies binding TNF may be used for personalized therapy of TNF-dependent diseases as a tool to predict the response to anti-TNF treatment. We generated recombinant fluorescent proteins consisting of the anti-TNF module based on the variable heavy chain (VHH) of camelid antibodies fused with the far-red fluorescent protein Katushka (Kat). Two types of anti-TNF VHH were developed: one (BTN-Kat) that was bound both human or mouse TNF, but did not neutralize their activity, and a second (ITN-Kat) that was binding and neutralizing human TNF. BTN-Kat does not interfere with TNF biological functions and can be used for whole-body imaging. ITN-Kat can be evaluated in humanized mice or in cells isolated from humanized mice. It is able to block human TNF (hTNF) activities both in vitro and in vivo and may be considered as a prototype of a theranostic agent for autoimmune diseases.

## 1. Introduction

Therapeutic neutralization of the inflammatory cytokines, in particular TNF, has revolutionized the treatment of autoimmune diseases including rheumatoid arthritis (RA), Crohn’s disease, spondyloarthritis (SpA), and others. However, a significant number of the patients (~50%) with RA and SpA do not respond, showing only marginal improvement, or initially responding but then relapsing [1,2,3,4]. Molecular imaging may be employed to investigate the mechanisms of the disease pathogenesis [5]. Several attempts to monitor the disease activity and the localization of therapeutic antibodies in the inflamed joints using molecular imaging with radiolabeled monoclonal anti-TNF antibodies (certolizumab pegol, adalimumab, and infliximab) in rheumatic diseases were reported [6,7,8,9,10]. At the same time, real-time images could be captured in vivo using a fairly simple equipment and appropriate fluorescent proteins [11]. The use of far-red fluorescent proteins, such as Katushka [12], allows for in vivo imaging of fluorescence in the deep tissues. To be able to visualize TNF in living tissue, fluorescent proteins can be genetically fused to a binding moiety, so that the resultant protein meets three criteria: (1) stable folding, (2) small size, and (3) affinity that is sufficient for specific antigen binding [13].

Since 1989, when a novel type of antibody devoid of the light chains was identified in the sera of various members of the *Camelidae* family [14], a number of therapeutic proteins and tools based on the variable heavy chain (VHH) of camelid antibodies were developed and evaluated. VHHs are the smallest functional antigen-binding domains of these heavy-chain-only antibodies, which are also called “nanobodies”. Their distinctive features are a small size, good stability and solubility, and high levels of expression in bacterial systems [15]. Also, it was shown that nanobodies are able to bind the epitopes inaccessible for conventional antibodies [16,17] and could display binding affinities in the lower nanomolar or even picomolar range [18]. All these features made nanobodies the ideal modules to be used in genetically encoded fluorescent proteins for successful molecular imaging. Several imaging techniques such as SPECT, PET, optical imaging, and ultrasound were successfully employed for nanobody-based imaging [19,20]. Nanobodies fused to fluorescent proteins were termed “chromobodies” and were used to visualize endogenous cellular structures in real-time studies of live cellular processes [21].

In this study two fluorescent sensors specific to TNF, both fused to far-red protein Katushka, were evaluated. One is based on VHH derived from Bactrian camel [22] and binds TNF without interfering with its functions. Therefore, it can be used to study the role of TNF in both normal and pathological conditions. The other protein is based on llama VHH, which binds and neutralizes human TNF bioactivity and can be used for the experimental therapy of the TNF-dependent autoimmune conditions in humanized mice with simultaneous visualization of the pathological processes in real time.

## 2. Materials and Methods

### 2.1. Design, Expression, and Purification of BTN-Kat and ITN-Kat Recombinant Proteins

DNA fragments encoding single-domain anti-TNF antibodies VHH41 [23] or ahTNF-VHH (GenBank: KU695528.1) [24,25] were cloned into the expression vector pET-28b (Merck Millipore, Darmstadt, Germany) between restriction sites BamHI and NcoI. To assemble the fluorescent-binding TNF nanobody (BTN-Kat) and the fluorescent-inhibiting TNF nanobody (ITN-Kat), the corresponding expression vector (which contained VHH41 or ahTNF-VHH, respectively) was digested with BamHI and NcoI and then ligated to PCR-amplified DNA fragment containing far-red fluorescent protein Katushka (TurboFP635, excitation/emission maxima at 588/635 nm) excised by Xhol- NcoI and DNA fragment encoded flexible glycine-serine linker. The C-terminal 6XHis tag sequence was in the same reading frame as the rest of the cDNA (Figure S1). Detailed information about expression and purification of the proteins is provided in Supplemental Material (Method S1, Table S1, Result S1).

### 2.2. Size Exclusion Chromatography

ITN-Kat (5 mg/mL) or BTN-Kat (5 mg/mL) was applied to a Superose 6 column (GE Healthcare, Amersham, UK) equilibrated in gel-filtration buffer (20 mM NaPi, pH 7.4, 150 mM NaCl). The flow rate was kept at 0.5 mL/min. Experimental and standard proteins were solubilized in gel-filtration buffer. The Superose 6 column was calibrated with standard proteins (Protein Standard Mix 15–600 kDa, Sigma-Aldrich, St. Louis, MO, USA). Elution volumes of ITN-Kat were found to be 12 mL, 4 mL, and 11 mL, and those of BTN-Kat were 11 mL, 3 mL, and 10.9 mL. To estimate their molecular masses, we used the plot of log_10_ molecular mass against elution volume. The same data were used to calculate the predicted molecular mass of the complex using the plot of molecular mass against elution volumes.

### 2.3. Mice

Human TNF knock-in mice (huTNFKI) described earlier [26,27] were bred in the SPF animal facility in the Institute of Biology and Biomedicine, Lobachevsky State University, in Nizhniy Novgorod on 12-h light/dark cycle at room temperature. All animal procedures were approved by the Scientific Council of the Institute of Biology and Biomedicine, Lobachevsky State University.

### 2.4. ELISA Measurement of the TNF Concentration in Murine Blood

Blood was taken from the buccal sinus of mice using sterile medical needles. To isolate the serum, blood was incubated at room temperature for 20–30 min and centrifuged at +4 °C and 14,000 rpm in clot activator tubes (Becton Dickinson, Franklin Lakes, NJ, USA). After that, the serum was transferred to fresh test tubes. TNF concentration was measured in sera by the Human-TNF ELISA Ready-SET-Go® (Fisher Scientific, Hampton, NH, USA).

### 2.5. Cytotoxic Assay

The inhibiting activity of BTN-Kat and ITN-Kat in TNF-mediated cytotoxicity was analyzed on WEHI-164 cell line [28]. The cells were plated at 5000 cells/well in 96-well culture plates. Recombinant hTNF was added at constant concentration (100 U/mL). The fluorescent antibodies were applied at serial dilutions 1 mM–2 pM. After 24 h of incubation, 3-(4,5-dimethylthiazol-2-yl)-2,5-diphenyltetrazolium bromide (MTT) (Sigma-Aldrich, St. Louis, MO, USA) was added at concentration 4 µg/mL. After 4 h of incubation, formazan crystals were solubilized in 10% *w*/*v* SDS solution in DMSO, and OD was measured at 560 nm. The percentage of living cells was calculated and fitted to a nonlinear regression curve using Prism 5 (GraphPad Software, San Diego, CA, USA) software. One unit was defined as the amount of TNF inhibitor sufficient to mediate half-maximal protection from cytotoxicity in the presence of 100 U/mL human TNF.

### 2.6. LPS/d-Galactosamine-Induced Acute Hepatotoxicity Model

TNF-humanized mice were individually weighted and received intraperitoneal injection of 150, 300, or 450 pmol/g of BTN-Kat; 75, 150, or 300 pmol/g of ITN-Kat; or 150 pmol/g of infliximab or PBS buffer followed 30 min later by an otherwise lethal dose of lipopolysaccharide and d-Galactosamine (LPS/D-Gal) (Sigma-Aldrich, St. Louis, MO, USA) 400 ng/g and 800 μg/g, respectively. Mice were euthanized when moribund. Kaplan–Meier survival curves were plotted, and pairwise statistical comparison of BTN-Kat, ITN-Kat, and infliximab was performed.

### 2.7. Flow Cytometry Analysis of Bone Marrow-Derived Macrophages

Bone marrow-derived macrophages (BMDM) from TNF-humanized mice or from C57Bl/6 mice were cultured for 10 days in DMEM, L-glutamune, Pen/Strep, 20% (*v*/*v*) horse serum, and 30% (*v*/*v*) of L929 conditioned medium, and then detached with ice-cold PBS and counted. Activation of TNF production by the macrophages was performed using LPS (100 ng/mL, 4 h, 37 °C, 5%, CO_2_). The cells mortality was obtained by measuring the percentage of dead macrophages by Trypan blue exclusion. Fcγ receptor was blocked by anti-Fcγ receptor antibodies (Biolegend, San Diego, CA, USA), and then cells were stained with anti-F4/80-FITC antibodies (Thermo Fischer, Waltham, MA, USA) and BTN-Kat or ITN-Kat by intracellular cytokine staining protocol using BD Fixation and Permeabilization Solution Kit with BD GolgiPlug™ (Becton Dickinson, Franklin Lakes, NJ, USA). The samples were analyzed on Cytoflex S flow cytometer (Beckman Coulter, Brea, CA, USA). The fluorescent signal from Katushka was detected with 585 nm laser excitation and 660/20 nm emission filter. Twenty thousand cells were evaluated per test; in a list mode, data were analyzed using CytExpert 2.0 software (Beckman Coulter, Brea, CA, USA).

### 2.8. Flow Cytometry Analysis of Murine Blood

Blood, collected from buccal sinus and treated with heparin (10 U/mL), was incubated with 1× RBC lysis buffer (Biolegend, San Diego, CA, USA) and then was resuspended in 100 μL of FACS buffer (PBS with 2% FBS), with Fixable Live/Dead Stain (Life Technologies, Carlsbad, CA, USA) and an appropriate combination of fluorescent antibodies specific to CD45, F4/80, CD3, CD45R (Thermo Fischer, Waltham, MA, USA), and FcR block (Biolegend, San Diego, CA, USA). After that, cells were incubated with BTN-Kat and ITN-Kat on ice without permeabilization, or stained by intracellular cytokine staining protocol using BD Fixation and Permeabilization Solution Kit with BD GolgiPlug™ (Becton Dickinson, Franklin Lakes, NJ, USA). Data were analyzed using CytExpert 2.0 (Beckman Coulter, Brea, CA, USA).

### 2.9. Fluorescence Whole-Body Imaging

150 pmol/g of the fluorescent sensors were injected intraperitonealy (i/p), and after 30 minu animals were i/p injected with LPS (400 ng/g) and d-Gal (800 μg/g) or the same volume of PBS. As a control, mice were injected with PBS 30 min prior to the injection of LPS/d-Gal instead of the proteins or the PBS only. Fluorescence imaging was performed on the IVIS-Spectrum system (Caliper Life Sciences, Waltham, MA, USA) in the epi-luminescence mode; the fluorescence was excited at 605/30 nm and detected at 660/20 nm [29]. Before the procedure, the mice were shaven using a shaving machine and additionally depilated with the cream. In 1, 3, and 6 h after the injection of LPS/ d-Gal, the mice were euthanized by isoflurane, and whole-body fluorescence images of the animals were acquired. Quantitative analysis was performed in the Living Image Software 4.2 (PerkinElmer, Waltham, MA, USA) by calculation of the fluorescence intensity averaged over the abdominal region and normalizing to the values of autofluorescence measured from the mice injected with PBS alone. To analyze the uptake of the BTN-Kat or ITN-Kat by the liver, ex vivo fluorescence imaging was performed.

### 2.10. Statistical Analysis

Statistically significant differences of values were determined using STATISTICA 10 (StatSoft, Moscow, Russia) using the Mann–Whitney U-test. Differences were considered statistically significant at *p* ≤ 0.05.

## 3. Results

### 3.1. Anti-TNF Antibodies Fused to Far-Red Katushka Protein Form Oligomers

**Figure 1:**
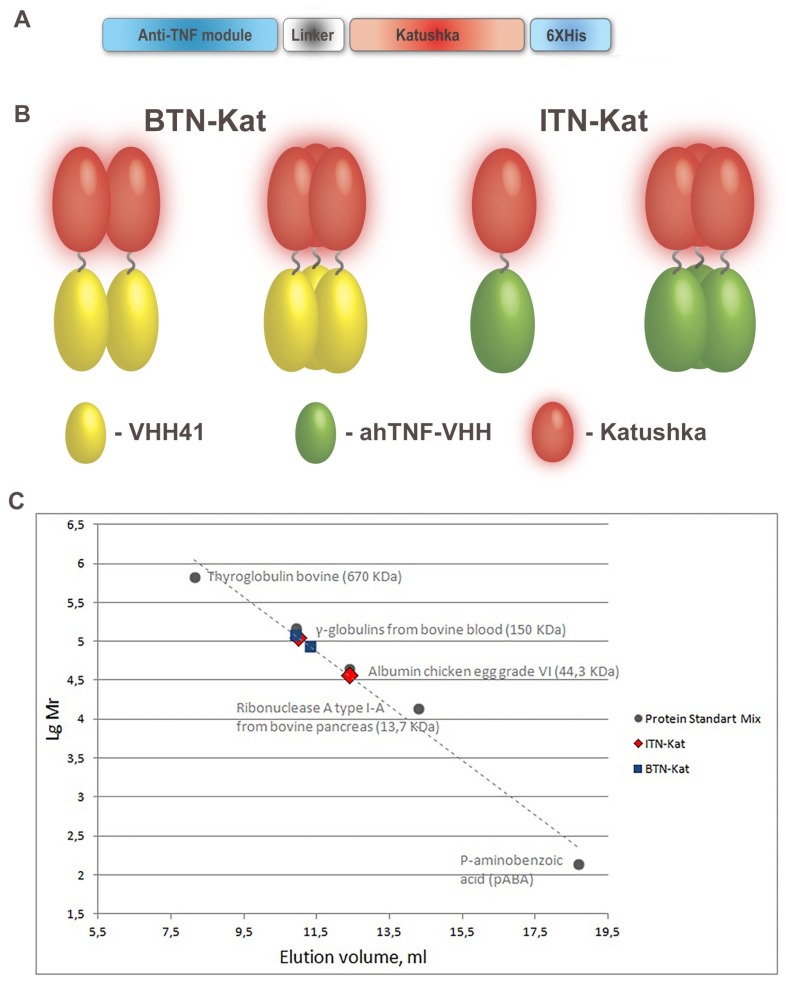
Fluorescent far-red anti-tumor necrosis factor (TNF) nanobodies spontaneously form oligomers. (**A**) Map of genetic constructs that encode fluorescent TNF sensors; (**B**) expected structure of BTN-Kat and ITN-Kat proteins in native conditions; (**C**) profile of size exclusion chromatography of ITN-Kat and BTN-Kat, imposed on the chromatogram of molecular weight markers (protein standard mix).

Two genetically encoded fluorescent sensors based on two distinct TNF-binding modules and far-red protein Katushka (Figure 1A) were designed and successfully expressed in prokaryotic system (Result S1, Figure S2). Native electrophoresis of purified proteins showed that both proteins formed a single structure under native conditions (Figure S3). Profiles from size exclusion chromatography showed that fluorescent proteins had a tendency to form oligomers (Figure 1C). Apparent molecular weight values of 113,260 kDa and 36,413 kDa for ITN-Kat corresponded to trimeric and monomeric forms, respectively. For BTN-Kat, apparent MW values were 85,979 kDa and 118,904 kDa, which corresponded to the dimeric and trimeric proteins (Figure 1C). Previously the ability of the fluorescent protein Katushka to form dimers and tetramers were reported [12,30], thus, we believe that oligomerization is due to the presence of the Katushka module. However, in our experiments a trimeric form for both proteins was also observed. The mixture of the protein’s oligomer forms was used for all subsequent experiments.

### 3.2. Fluorescent Nanobodies Interact with TNF In Vitro

**Figure 2:**
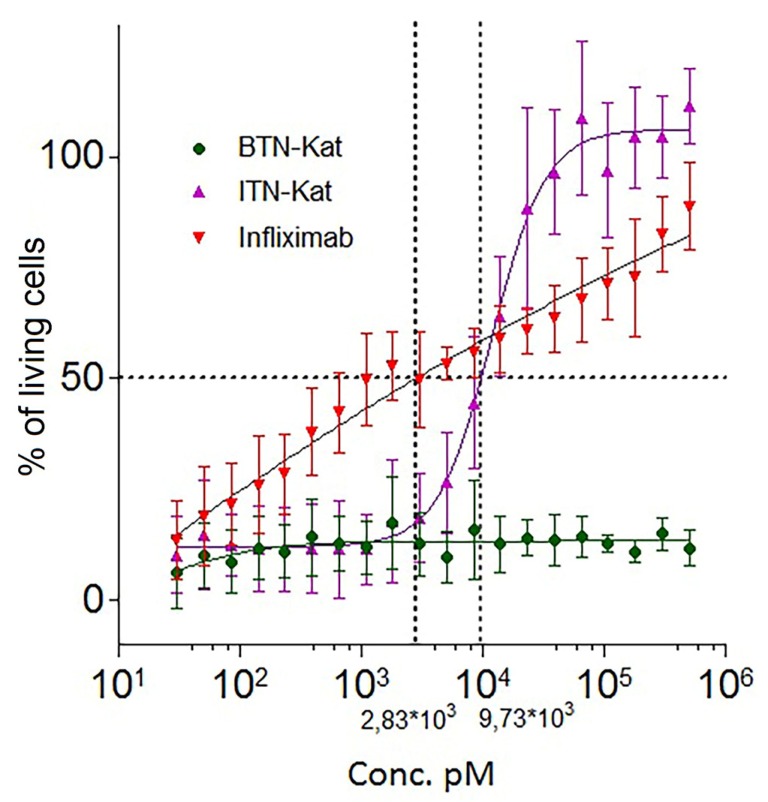
ITN-Kat, but not BTN-Kat, inhibited human TNF (hTNF) activity in vitro. TNF-neutralizing activities of BTN-Kat, ITN-Kat, and infliximab were evaluated by MTT cytotoxicity test using the WEHI 164 cell line. Percentage of living cells ±SD is plotted. Dashed lines indicate half maximal effective concentration (EC_50_)

Inhibitory activities of BTN-Kat and ITN-Kat toward recombinant human TNF were evaluated in TNF-dependent colorimetric MTT cytotoxicity assay with WEHI 164 murine fibrosarcoma cell line, which is sensitive to human TNF. The analysis was prepared in 96-well plates. Each well contained 20,000 cells, the concentration of TNF was 100 U/mL in accordance with a predetermined TNF DL50 (1U). Cells were incubated with a mixture of TNF and inhibitor in a range of concentrations overnight. In contrast to BTN-Kat, ITN-Kat demonstrated a dose-dependent hTNF inhibitory activity (half maximal effective concentration (EC_50_) = 9.73·10^3^ pM). The clinically utilized chimeric monoclonal antibody infliximab was used as a positive control (EC_50_ = 2.83·10^3^ pM) (Figure 2).

**Figure 3:**
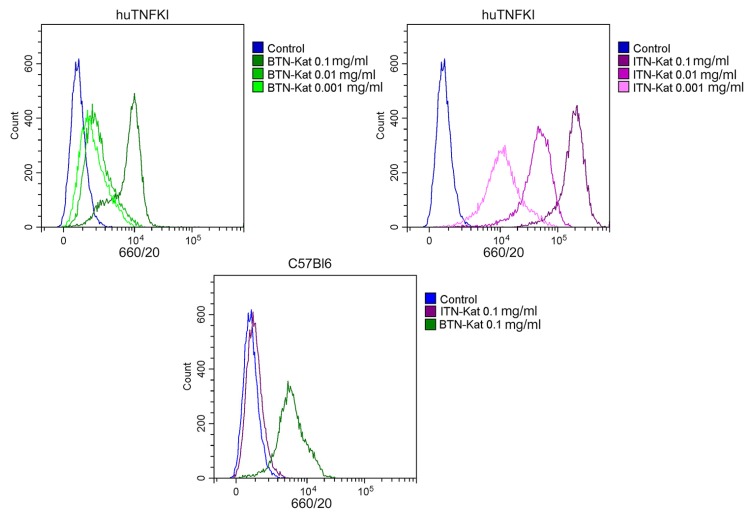
ITN-Kat is specific for human TNF while BTN-Kat interacts with both human and murine TNF. (**A**) BTN-Kat and ITN-Kat binding activity to bone marrow-derived macrophages (BMDM) from TNF-humanized (huTNFKI) mice. (**B**) BTN-Kat binding activity to BMDM from C57Bl6 mice (WT). Macrophages were activated by lipopolysaccharide (LPS) (100 ng/mL for 4 h) in the presence of brefeldin A and stained for TNF by BTN-Kat or ITN-Kat using intracellular cytokine staining protocol. Shown cells are gated on F4/80 expression.

Additionally, in vitro TNF binding activity of BTN-Kat and ITN-Kat was examined using flow cytometry. Bone marrow-derived macrophages (BMDM) derived from TNF humanized mice were activated by bacterial LPS and then stained with BTN-Kat and ITN-Kat using an intracellular cytokine staining protocol that resulted in specific staining of the TNF-expressing cells. BTN-Kat demonstrated lower staining than ITN-Kat in BMDM cells from huTNFKI mice (Figure 3A); however, BTN-Kat was also able to interact with mouse TNF derived from the BMDM of C57Bl6 mice while ITN-Kat did not (Figure 3B). This observation suggested that imaging with BTN-Kat may be feasible in wild-type mice.

### 3.3. ITN-Kat Showed TNF Neutralizing Activity In Vivo, while BTN-Kat Did not

**Figure 4:**
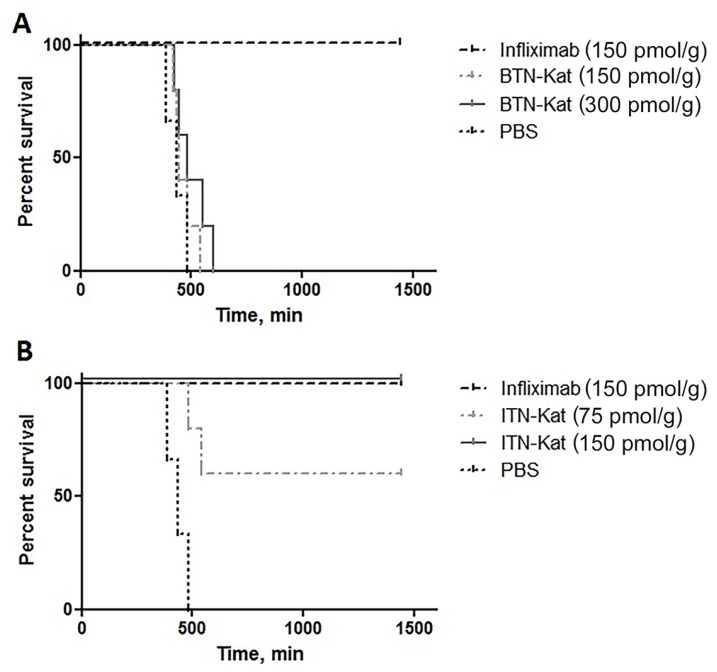
Protection from LPS/d-Galactoseamine (LPS/D-Gal) toxicity in vivo is provided by ITN-Kat, but not by BTN-Kat. TNF-humanized mice were injected either with ITN-Kat, BTN-Kat, infliximab, or PBS. Thirty minutes later, mice were injected with the otherwise lethal dose of LPS/d-Gal. (**A**) Survival curves of mice injected with 300 pmol/g and 150 pmol/g of BTN-Kat compared with the buffer or 150 pmol/g infliximab; (**B**) survival curves of mice injected with 150 pmol/g and 75 pmol/g of ITN-Kat as compared with the buffer or 150 pmol/g infliximab.

The ability of BTN-Kat and ITN-Kat to inhibit systemic TNF was evaluated in TNF-humanized mice in the experimental model of acute hepatotoxicity (Figure 4).

Mice injected with ITN-Kat at concentration 150 pmol/g survived (Figure 4B), as did mice treated with infliximab at the same dose. BTN-Kat was not able to protect mice from lethality at the same dose nor at higher doses (Figure 4A).

### 3.4. The In Vivo Fluorescence of Anti-TNF Nanobodies Correlates with TNF Levels in Mice

**Figure 5:**
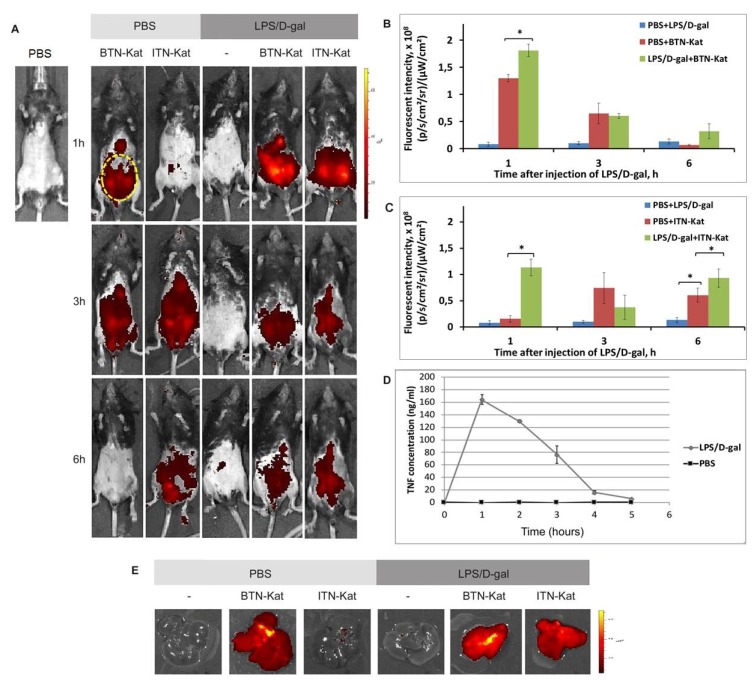
Fluorescent anti-TNF nanobodies accumulate in the liver of mice after the LPS/d-Gal injection. Visualization of TNF production in adult humanized TNF knock-in mice using BTN-Kat and ITN-Kat during LPS/d-Gal acute hepatotoxicity. (**A**) Fluorescence intensity images in mice during acute hepatotoxicity in comparison with the control; the abdominal region for quantitative analysis is displayed by the yellow circle; (**B**) fluorescence signal analysis of mice after injection of BTN-Kat followed by LPS/d-gal or PBS injection. Intensity of fluorescence ±SD is plotted; (**C**) fluorescence signal analysis of mice after injection of ITN-Kat followed by LPS/d-gal or PBS injection. Mean ± SD is plotted; (**D**) TNF level in mice serum after LPS/d-gal or PBS injection measured by ELISA; (**E**) fluorescence intensity ex vivo images of mice liver 1 h after LPS/d-gal injection.

TNF plays a critical role in liver injury induced by LPS/d-Gal. Thus, the model of acute hepatotoxicity is characterized by an inflammatory process in the abdominal cavity, and, as expected, the highest level of fluorescence of the sensors was detected in the abdominal area. The peak of the fluorescence intensity was observed at 1 h post-injection of the LPS/d-Gal. In this group fluorescence signal was significantly higher (*p* < 0.05) than in the control mice injected with PBS and LPS/d-Gal or PBS alone (Figure 5A–C). These results are consistent with the data obtained by ELISA, in which the highest level of TNF concentration in the blood was observed 1 h after the LPS/d-Gal injection (Figure 5D). However, at the 3-h time point after the LPS/d-Gal injection, the level of the BTN-Kat and ITN-Kat fluorescence did not differ from the mice injected with the sensor alone without LPS/d-Gal. At 6 h, the fluorescence intensity in mice injected with BTN-Kat fell to the baseline level of autofluorescence (Figure 5B), which suggested either complete excretion of BTN-Kat protein from the body or its degradation. However, at 6-h point, the fluorescent signals of control ITN-Kat-injected mice and ITN-Kat-injected mice challenged with LPS/d-Gal were significantly distinct from autofluorescence (*p* < 0.05) (Figure 5C). The results of ex vivo imaging confirmed the accumulation the fluorescent anti-TNF antibodies in the liver with the maximum of fluorescence signals at 1 h after LPS/d-Gal injection (Figure 5E).

### 3.5. LPS/d-Galactosamine-Induced Acute Hepatotoxicity Depends on TNF Expression by Monocytes

**Figure 6:**
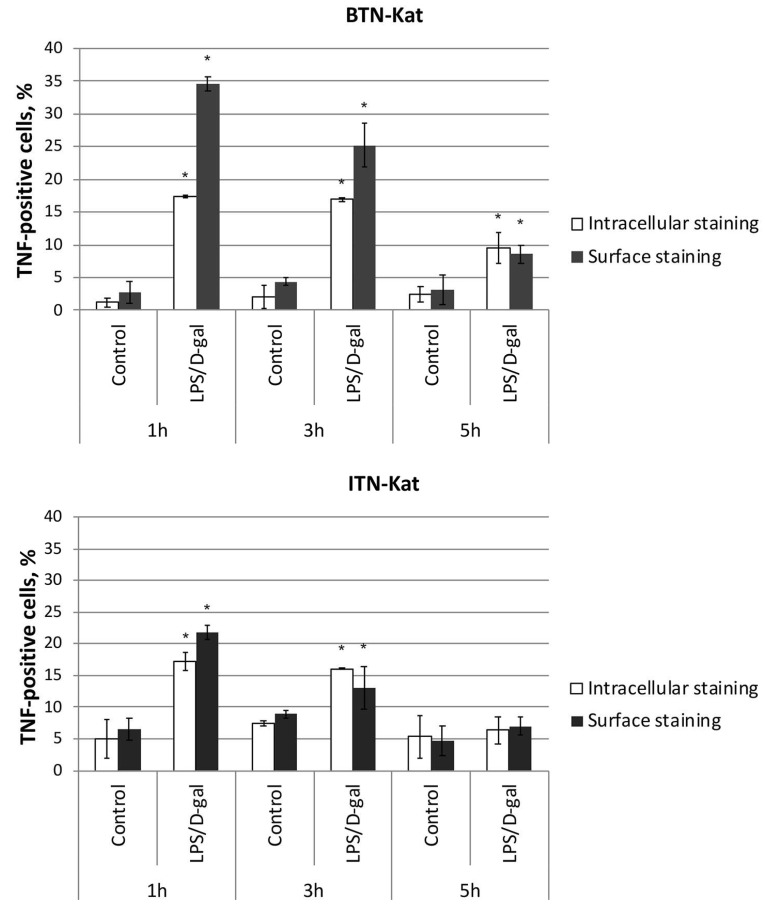
F4/80-positive cells are the main source of TNF. Levels of TNF-positive F4/80-positive cells 1 h, 3 h, and 5 h after LPS/d-gal injection were measured using BTN-Kat and ITN-Kat by flow cytometry surface and intracellular staining protocols. Mean levels of TNF-positive cells ±SD are plotted. Data are representative of five independent experiments. (* *p* < 0.05).

We then evaluated the expression levels of TNF by F4/80, CD3, and CD45R-positive cells (Figure 6 and Figure S5) using flow cytometry protocols for both surface and intracellular staining during LPS/d-gal-induced acute hepatotoxicity. The results showed that the main source of TNF corresponded to F4/80-positive cells. At the same time, the maximal number of TNF-positive cells was observed at 1 h after the LPS/d-gal injection, in correlation with the TNF level in the blood (Figure 5D). Of note, we did not observe any significant changes in the TNF levels produced by other cell types (Figure S5).

## 4. Discussion

Molecular imaging is a promising approach to address the role of TNF in various inflammatory pathologies. Recent studies highlighted applications of red fluorescent proteins fused with VHH antibodies for bioimaging and theranostics [31,32,33]. The small size and the absence of Fc-fragment in VHH modules may reduce side effects caused by interactions with receptors of immunocompetent cells and with complement system, as it usually happens with classical antibodies [34]. Also, the earlier study [35] demonstrated that red fluorescent proteins with maximum of emission spectra >600 nm are more effective, because the emitted light is not absorbed by the tissues.

We attempted to utilize far-red-emitting nanobody-based fusion proteins for imaging both systemic TNF as well as TNF at the sites of its local expression in vivo. VHH domains from *Camelidae* antibodies VHH41 [23] and ahTNF-VHH [24] were used as targeting modules. It has been shown that those module had a similar affinity to hTNF, however VHH41’s target region of hTNF was not involved in interaction with the TNF receptor [23]. Fluorescent protein Katushka chosen as the imaging molecule emits in the range of 620–660 nm that fits the window of biological tissue transparency in which case the absorption is minimal [35]. Moreover, Katushka was characterized by a very high pH and photostability, and by intensity of the signal 7- to 10-fold brighter than the spectrally close HcRed [36] or mPlum [37] proteins. Additionally, it readily forms tetrameric structures at pH 5.5 to 8.5, but can dissociate into the dimer at pH below 5.0 [30]. We noticed that proteins fused with Katushka also showed a tendency to form oligomeric structures (Figure 1C). The other advantage of the fluorescent complexes “VHH-Katushka” is their relatively simple and sufficiently effective expression in prokaryotic systems (Method S1, Result S1).

In human TNF knock-in (huTNFKI) mice [26] used here, human TNF was expressed in vivo instead of murine TNF. It mediated normal and pathological functions of this cytokine which can be neutralized by clinically used anti-hTNF drugs. One of the studied nanobodies, ITN-Kat, was able to bind to human TNF (Figure 3) and neutralize its activity in vitro (Figure 2) as well as in vivo (Figure 4B). The protective effect of ITN-Kat was confirmed by liver histology (Result S2, Figure S4E). On the other hand, although BTN-Kat can bind both human and murine TNF (Figure 3), it lacks blocking ability (Figure 2) and therefore could not prevent the development of acute hepatotoxicity (Figure 4A, Figure S4D). Moreover, ITN-Kat showed higher sensitivity to hTNF than BTN-Kat. We observed a higher level of fluorescence in activated macrophages stained with ITN-Kat compared to BTN-Kat in a similar concentration range using flow cytometry (Figure 3A).

As a systemic reaction, septic shock affects all body systems, including immune cells. TNF is known to play a critical role in the process of liver injury induced by LPS/d-Gal. Soluble TNF is the main hepatotoxic mediator in this toxicity model [38]. Using fluorescent sensors targeting TNF, we confirmed that the main source of TNF during the development of LPS/d-gal-induced acute hepatotoxicity is the myeloid cell compartment (Figure 6) [39]. More specifically, our results indicate that TNF expressed by F4/80-positive cells plays the key role in the development of pathology, while T-cells and B-cells do not contribute to the increase of serum TNF levels in the process of acute inflammation (Figure S5).

The inflammatory processes in the abdominal cavity of humanized mice after LPS/d-gal administration were studied using BTN-Kat and ITN-Kat in whole-body and ex vivo imaging mode. The peak of fluorescence at the time of maximal concentration of TNF in the blood occurred 1 h after LPS/d-gal. intraperitoneal injection, consistent with the data obtained by ELISA (Figure 5D). Furthermore, the level of BTN-Kat fluorescence gradually decreased, and this paralleled the level of TNF in the blood. The elevated expression of TNF in joints in the murine model of collagen-induced arthritis was successfully visualized by BTN-Kat [40]. These data confirmed the ability of BTN-Kat to bind both human and murine TNF, with subsequent successful visualization using bioimaging methods. The level of ITN-Kat fluorescence did not correlate with the level of TNF in the blood during acute hepatotoxicity. This may indicate that this TNF inhibitor affected regulation of TNF expression since cytokine gene regulation may include positive feedback loops. This hypothesis requires additional experimental evaluation.

In summary, we developed and characterized TNF fluorescent sensor (BTN-Kat) and fluorescent sensor-inhibitor (ITN-Kat), utilizing two single-domain anti-TNF antibodies. We evaluated their ability to bind and neutralize TNF in vitro, and to serve as imaging labels in vitro and in the whole body non-invasive analysis. We concluded that BTN-Kat is a convenient tool for studying the dynamics of TNF expression without interfering with its biological functions, while ITN-Kat is a prototype theranostic agent for TNF-dependent autoimmune diseases.

## Supplementary Materials

The following are available online at http://www.mdpi.com/2073-4468/7/4/43/s1, Figure S1: Protein sequences of BTN-Kat and ITN-Kat proteins; Figure S2: The yields of the BTN-Kat and ITN-Kat proteins purified from the various *E. coli* strains; Figure S3: Native electrophoresis of BTN-Kat and ITN-Kat; Figure S4: Liver histology (H&E stain) of mice; Figure S5: Levels of TNF-positive CD3 and CD45R-positive cells; Table S1: The *E. coli* strains used in this study; Method S1: Expression and purification of BTN-Kat and ITN-Kat recombinant proteins; Method S2: Preparation of Liver Tissues for Histological Analysis; Result S1: Fluorescent antibodies expressed in BL21(DE3) strain of *E. coli*; Result S2: The therapeutic effect of BTN-Kat and ITN-Kat proteins.

## References

[1] Davis J., van der Heijde D.M., Braun J., Dougados M., Cush J., Clegg D., Inman R.D., Kivitz A., Zhou L., Solinger A. (2005). Sustained durability and tolerability of etanercept in ankylosing spondylitis for 96 weeks. Ann. Rheum. Dis..

[2] Van der Heijde D., Dijkmans B., Geusens P., Sieper J., DeWoody K., Williamson P., Braun J., Ankylosing Spondylitis Study for the Evaluation of Recombinant Infliximab Therapy Study Group (2005). Efficacy and safety of infliximab in patients with ankylosing spondylitis: Results of a randomized, placebo-controlled trial (ASSERT). Arthritis Rheum..

[3] Van der Heijde D., Kivitz A., Schiff M.H., Sieper J., Dijkmans B.A., Braun J., Dougados M., Reveille J.D., Wong R.L., Kupper H., Davis J.C., ATLAS Study Group (2006). Efficacy and safety of adalimumab in patients with ankylosing spondylitis: Results of a multicenter, randomized, double-blind, placebo-controlled trial. Arthritis Rheum..

[4] Inman R.D., Davis J.C., Heijde Dv., Diekman L., Sieper J., Kim S.I., Mack M., Han J., Visvanathan S., Xu Z., Hsu B. (2008). Efficacy and safety of golimumab in patients with ankylosing spondylitis: Results of a randomized, double-blind, placebo-controlled, phase III trial. Arthritis Rheum..

[5] Kuchmiy A.A., Efimov G.A., Nedospasov S.A. (2012). Methods for in vivo molecular imaging. Biochemistry.

[6] Palframan R., Airey M., Moore A., Vugler A., Nesbitt A. (2009). Use of biofluorescence imaging to compare the distribution of certolizumab pegol, adalimumab, and infliximab in the inflamed paws of mice with collagen-induced arthritis. J. Immunol. Methods.

[7] Malviya G., Conti F., Chianelli M., Scopinaro F., Dierckx R.A., Signore A. (2010). Molecular imaging of rheumatoid arthritis by radiolabelled monoclonal antibodies: new imaging strategies to guide molecular therapies. Eur. J. Nucl. Med. Mol. Imaging.

[8] Lambert B., Carron P., D’Asseler Y., Bacher K., van den Bosch F., Elewaut D., Verbruggen G., Beyaert R., Dumolyn C., De Vos F. (2016). (99m)Tc-labelled S-HYNIC certolizumab pegol in rheumatoid arthritis and spondyloarthritis patients: a biodistribution and dosimetry study. EJNMMI Res..

[9] Carron P., Lambert B., Van Praet L., De Vos F., Varkas G., Jans L., Elewaut D., Van den Bosch F. (2016). Scintigraphic detection of TNF-driven inflammation by radiolabelled certolizumab pegol in patients with rheumatoid arthritis and spondyloarthritis. RMD Open.

[10] Put S., Westhovens R., Lahoutte T., Matthys P. (2014). Molecular imaging of rheumatoid arthritis: emerging markers, tools, and techniques. Arthritis Res. Ther..

[11] Hoffman R.M. (2016). Use of fluorescent proteins and color-coded imaging to visualize cancer cells with different genetic properties. Cancer Metast. Rev..

[12] Shcherbo D., Merzlyak E.M., Chepurnykh T.V., Fradkov A.F., Ermakova G.V., Solovieva E.A., Lukyanov K.A., Bogdanova E.A., Zaraisky A.G., Lukyanov S. (2007). Bright far-red fluorescent protein for whole-body imaging. Nat. Methods.

[13] Kaiser P.D., Maier J., Traenkle B., Emele F., Rothbauer U. (2014). Recent progress in generating intracellular functional antibody fragments to target and trace cellular components in living cells. Biochim. Biophys. Acta.

[14] Hamers-Casterman C., Atarhouch T., Muyldermans S., Robinson G., Hamers C., Songa E.B., Bendahman N., Hamers R. (1993). Naturally occurring antibodies devoid of light chains. Nature.

[15] Olichon A., Surrey T. (2007). Selection of Genetically Encoded Fluorescent Single Domain Antibodies Engineered for Efficient Expression in Escherichia coli. J. Biol. Chem..

[16] Desmyter A., Transue T.R., Ghahroudi M.A., Thi M.H., Poortmans F., Hamers R., Muyldermans S., Wyns L. (1996). Crystal structure of a camel single-domain VH antibody fragment in complex with lysozyme. Nat. Struct. Biol..

[17] Muyldermans S., Cambillau C., Wyns L. (2001). Recognition of antigens by single-domain antibody fragments: the superfluous luxury of paired domains. Trends Biochem. Sci..

[18] van der Linden R.H.J., Frenken L.G., de Geus B., Harmsen M.M., Ruuls R.C., Stok W., de Ron L., Wilson S., Davis P., Verrips C.T. (1999). Comparison of physical chemical properties of llama VHH antibody fragments and mouse monoclonal antibodies. Biochim. Biophys. Acta.

[19] Chakravarty R., Goel S., Cai W. (2014). Nanobody: The “Magic Bullet” for Molecular Imaging?. Theranostics.

[20] Rashidian M., Keliher E.J., Bilate A.M., Duarte J.N., Wojtkiewicz G.R., Jacobsen J.T., Cragnolini J., Swee L.K., Victora G.D., Weissleder R., Ploegh H.L. (2015). Noninvasive imaging of immune responses. Proc. Natl. Acad. Sci. USA.

[21] Rothbauer U., Zolghadr K., Tillib S., Nowak D., Schermelleh L., Gahl A., Backmann N., Conrath K., Muyldermans S., Cardoso M.C. (2006). Targeting and tracing antigens in live cells with fluorescent nanobodies. Nat. Methods.

[22] Tillib S.V., Vyatchanin A.S., Muyldermans S. (2014). Molecular analysis of heavy chain-only antibodies of Camelus bactrianus. Biochemistry.

[23] Efimov G.A., Khlopchatnikova Z.V., Sazykin A.Yu., Drutskaya M.C., Kruglov A.A., Shilov E.C., Kuchmii A.A., Nedospasov S.A., Tillib S.V. (2012). Isolation and characteristics of a new recombinant single domain antibody that specifically binds to human TNF. Russ. J. Immunol..

[24] Coppieters K., Dreier T., Silence K., de Haard H., Lauwereys M., Casteels P., Beirnaert E., Jonckheere H., Van de Wiele C., Staelens L., Hostens J. (2006). Formatted anti-tumor necrosis factor alpha VHH proteins derived from camelids show superior potency and targeting to inflamed joints in a murine model of collagen-induced arthritis. Arthritis Rheum..

[25] Plagmann I., Chalaris A., Kruglov A.A., Nedospasov S., Rosenstiel P., Rose-John S., Scheller J. (2009). Transglutaminase-catalyzed covalent multimerization of Camelidae anti-human TNF single domain antibodies improves neutralizing activity. J. Biotechnol..

[26] Olleros M.L., Chavez-Galan L., Segueni N., Bourigault M.L., Vesin D., Kruglov A.A., Drutskaya M.S., Bisig R., Ehlers S., Aly S., Walter K. (2015). Control of Mycobacterial Infections in Mice Expressing Human Tumor Necrosis Factor (TNF) but Not Mouse TNF. Infect Immun..

[27] Kruglov A.A., Tumanov A.V., Grivennikov S.I., Shebzukhov Y.V., Kuchmiy A.A., Efimov G.A., Drutskaya M.S., Scheller J., Kuprash D.V., Nedospasov S.A. (2011). Modalities of Experimental TNF Blockade In Vivo: Mouse Models.

[28] Espevik T., Nissen-Meyer J. (1986). A highly sensitive cell line, WEHI 164 clone 13, for measuring cytotoxic factor/tumor necrosis factor from human monocytes. J. Immunol. Methods.

[29] Yuzhakova D.V., Shirmanova M.V., Bocharov A.A., Astrakhantseva I.V., Vasilenko E.A., Gorshkova E.N., Drutskaya M.S., Zagaynova E.V., Nedospasov S.A., Kruglov A.A. (2016). Microbiota Induces Expression of Tumor Necrosis Factor in Postnatal Mouse Skin. Biochemistry.

[30] Pletneva N.V., Pletnev V.Z., Shemiakina I.I., Chudakov D.M., Artemyev I., Wlodawer A., Dauter Z., Pletnev S. (2011). Crystallographic study of red fluorescent protein eqFP578 and its far-red variant Katushka reveals opposite pH-induced isomerization of chromophore. Protein Sci..

[31] Kijanka M., Dorresteijn B., Oliveira S., van Bergen en Henegouwen P.M. (2015). Nanobody-based cancer therapy of solid tumors. Nanomedicine.

[32] Albert S., Arndt C., Koristka S., Berndt N., Bergmann R., Feldmann A., Schmitz M., Pietzsch J., Steinbach J., Bachmann M. (2018). From mono- to bivalent: improving theranostic properties of target modules for redirection of UniCAR T cells against EGFR-expressing tumor cells in vitro and in vivo. Oncotarget.

[33] Van Brussel A.S.A., Adams A., Oliveira S., Dorresteijn B., El Khattabi M., Vermeulen J.F., van der Wall E., Mali W.P., Derksen P.W., van Diest P.J., van Bergen En Henegouwen P.M. (2016). Hypoxia-Targeting Fluorescent Nanobodies for Optical Molecular Imaging of Pre-Invasive Breast Cancer. Mol. Imaging Biol..

[34] Muyldermans S. (2013). Nanobodies: Natural Single-Domain Antibodies. Ann. Rev. Biochem..

[35] Deliolanis N.C., Kasmieh R., Wurdinger T., Tannous B.A., Shah K., Ntziachristos V. (2008). Performance of the Red-shifted Fluorescent Proteins in deep-tissue molecular imaging applications. J. Biomed. Opt..

[36] Gurskaya N.G., Fradkov A.F., Terskikh A., Matz M.V., Labas Y.A., Martynov V.I., Yanushevich Y.G., Lukyanov K.A., Lukyanov S.A. (2001). GFP-like chromoproteins as a source of far-red fluorescent proteins. FEBS Lett..

[37] Wang L., Jackson W.C., Steinbach P.A., Tsien R.Y. (2004). Evolution of new nonantibody proteins via iterative somatic hypermutation. Proc. Natl. Acad. Sci. USA.

[38] Olleros M.L., Vesin D., Fotio A.L., Santiago-Raber M.L., Tauzin S., Szymkowski D.W., Garcia I. (2010). Soluble TNF, but not membrane TNF, is critical in LPS-induced hepatitis. J. Hepatol..

[39] Grivennikov S.I., Tumanov A.V., Liepinsh D.J., Kruglov A.A., Marakusha B.I., Shakhov A.N., Murakami T., Drutskaya L.N., Förster I., Clausen B.E. (2005). Distinct and nonredundant in vivo functions of TNF produced by T cells and macrophages/neutrophils: protective and deleterious effects. Immunity.

[40] Drutskaya M.S., Efimov G.A., Zvartsev R.V., Chashchina A.A., Chudakov D.M., Tillib S.V., Kruglov A.A., Nedospasov S.A. (2014). Experimental models of arthritis in which pathogenesis is dependent on tnf expression. Biochemistry.

