# Supplementary material for: Properties of Fluorescent Far-Red Anti-TNF Nanobodies
Source: Antibodies (Basel). 2018 Dec 15;7(4):43. doi: 10.3390/antib7040043 (PMC6698962; doi:10.3390/antib7040043)
Supplement: Supplementary file 1 [file antibodies-07-00043-s001.pdf]

Supplementary Materials:

```
ITN-Kat   MGSQVQLQESGGGLVQPGGSLRLSCAASGRTFSDHSGYTYTIGWFRQAPGKEREFVARIYWSSGNTYYADSVKGRFAISR    80

BTN-Kat   M-AQVQLVESGGGSVQAGGSLRLSCAASGSTNSELC-----MAWFREVPGKEREVVALVN-SDGRTIYGEAVKGRFTMSK    73


ITN-Kat   DIAKNTVDLTMNNLEPEDTAVYYCAARDGIPTSRSVESYNYWGQGTQVTVSSA-------------GAGSGGGGSGMVGE   147

BTN-Kat   DNAKNTLYLQMNSLKAEDTAMYYCAAGPGF-FGRC--NYNSVGQGTQVTVSSGRYPYDVPDYASGRGSGSGGGGSGMVGE   150


ITN-Kat   DSVLITENMHMKLYMEGTVNDHHFKCTSEGEGKPYEGTQTMKIKVVEGGPLPFAFDILATSFMYGSKTFINHTQGIPDFF   227

BTN-Kat   DSVLITENMHMKLYMEGTVNDHHFKCTSEGEGKPYEGTQTMKIKVVEGGPLPFAFDILATSFMYGSKTFINHTQGIPDFF   230


ITN-Kat   KQSFPEGFTWERITTYEDGGVLTATQDTSLQNGCLIYNVKINGVNFPSNGPVMQKKTLGWEASTEMLYPADSGLRGHAQM   307

BTN-Kat   KQSFPEGFTWERITTYEDGGVLTATQDTSLQNGCLIYNVKINGVNFPSNGPVMQKKTLGWEASTEMLYPADSGLRGHAQM   310


ITN-Kat   ALKLVGGGYLHCSLKTTYRSKKPAKNLKMPGFYFVDRRLERIKEADKETYVEQHEMAVARYCDLPSKLGHSSGLRSLEHH   387

BTN-Kat   ALKLVGGGYLHCSLKTTYRSKKPAKNLKMPGFYFVDRRLERIKEADKETYVEQHEMAVARYCDLPSKLGHSSGLRSLEHH   390


ITN-Kat   HHHH                                                                              391

BTN-Kat   HHHH                                                                              394
```

Figure S1: Protein sequences of BTN-Kat and ITN-Kat proteins. Amino acids in green form the anti-TNF region (covered by patent RU2530553 in BTN-Kat and covered by patent US2007/0237769A1 in ITN-Kat) , in blue – linker region, in red – Katushka (TurboFP635) region, in grey – hexa histidine-tag.

Method S1: Expression and purification of BTN-Kat and ITN-Kat recombinant proteins.

All the expression and purification experiments were performed using *E. coli* DE3 strains, namely, Rosetta 2 pLysS, BL21, BL21 Codon plus, Lemo21, and B834. *E. coli* DE3 strains contain lambda DE3 lysogen and are intended to be used for strong expression of proteins encoded by plasmids with the T7 promoter. The B834(DE3) strain is a Met auxotroph. This strain widely used for highly specific [35S] methionine-labeling of target proteins, and recently, for selenomethionyl derivatization of proteins for X-ray crystallography [1]. BL21(DE3) strain is deficient in the OmpT and Lon proteases, which may interfere with the isolation of intact recombinant proteins [2]. Rosetta2(DE3) strain is a BL21(DE3) derivative which expresses

seven rare tRNAs and facilitates expression of genes that encode rare *E. coli* codons. It was designed to enhance the expression of eukaryotic proteins that contain codons rarely used in *E. coli*. The BL21-CodonPlus (DE3) is also a BL21(DE3) derivative and contains extra copies of the argU, ileY, and leuW, as well as the proL tRNA, genes. This strain rescues expression of heterologous proteins from organisms that have either AT- or GC-rich genomes. The other derivative strain of *E. coli* is BL21(DE3), termed Lemo21(DE3), in which the activity of the T7 RNA polymerase can be precisely controlled by its natural inhibitor T7 lysozyme [3]. Detailed information about the bacterial strains used in this study is provided in Table S1.

Table S1: The *E. coli* strains used in this study

The E.coli strains	Description	Source
B834(DE3)	F-ompT gal hsdSB (rB-mB-) met dcm lon λ DE3	Merck Millipore, Germany
BL21(DE3)	E. coli B F– dcm ompT hsdS(rB–mB–) gal λ(DE3)	Merck Millipore, Germany
Rosetta 2(DE3)	F–ompT hsdSB(rB–mB–) gal dcm(DE3) pLysS-RARE23(CamR)	Merck Millipore, Germany
BL21-CodonPlus (DE3)-RP	E. coli B F–ompT hsdS(rB–mB–) dcm+TetR gal endA Hte [argU proL CamR]	Agilent Technologies, USA
Lemo21(DE3)	fhuA2 [lon] ompT gal (λ DE3) [dcm] ΔhsdS/ pLemo(CamR)	New England Biolabs, Inc, USA

Bacterial culture was grown in the presence of a selective antibiotic to optical density of 0.6 at 37ºC with shaking at 200 rpm. After that, the culture was cooled on ice, and induction was performed using 0,1 mM isopropyl β-D-1-thiogalactopyranoside. Then, the bacterial culture was grown for additional 6 h at 30ºC with shaking at 200 rpm. The cells were centrifuged and resuspended in lysis buffer containing 50 mM Tris-HCl (pH 7), 300 mM NaCl, 5% glycerol, 0.5

% Triton X-100, 2.5 mM $MgCl_2$, 0.02 mg/ml lysozyme, and 0.1 mM PMSF. The cells were disintegrated by sonication. The cytoplasmic fraction was either immediately processed for protein purification or stored at −125ºC until use.

The supernatant was passed through Bio Scale Mini Profinity IMAC column (BioRad, USA), which was equilibrated in buffer 1, containing 50 mM NaPi (pH 8.5), 300 mM NaCl, 10 mM imidazole, and 1 mM 2-mercaptoethanol. The column was washed with 10 bed volumes of buffer 2 (50 mM NaPi (pH 8.5), 300 mM NaCl, 20 mM imidazole, and 1 mM 2-mercaptoethanol). The protein was eluted by buffer 3 (50 mM NaPi (pH 8.5), 300 mM NaCl, 250 mM imidazole, and 1 mM 2-mercaptoethanol). The eluted protein was dialyzed against PBS and concentrated using 2-ml VIVA SPIN concentrator (Sartorius Vivascience GmbH, Germany). After that, the proteins were treated by Triton-114 to purify them from LPS [4]. The purified proteins were filtered in sterile conditions before analysis. The final protein concentration was measured using the spectrophotometer Nanophotometer P-330 (Implen, Germany).

The obtained proteins were further analyzed by native (Figure S2) and SDS-page (data not shown) electrophoresis.

Method S2: Preparation of Liver Tissues for Histological Analysis. The liver was cut out of the mice upon death or 24 hours after LPS/D-Gal-induced acute hepatotoxicity. Liver tissues were kept for 3 days in 4% phosphate-buffered formalin solution. Then, each liver was cut into 1 cm thick slices, dehydrated with alcohol, cleared with xylol, and finally embedded in paraffin. Using microtome, serial paraffin sections of 4 μm thickness were made, and the sections were then stained with hematoxylin and eosin as previously described [5].

Result S1: Fluorescent antibodies expressed in BL21(DE3) strain of *E.coli*. Several *E.coli* strains were used to obtain the target proteins (Table S1). After the induction of the protein expression in 1L of the medium with the following purification, the yields of the target proteins were measured and compared (Figure S1). BL21 (DE3) was chosen as an expression strain for both proteins, because it was the most productive.

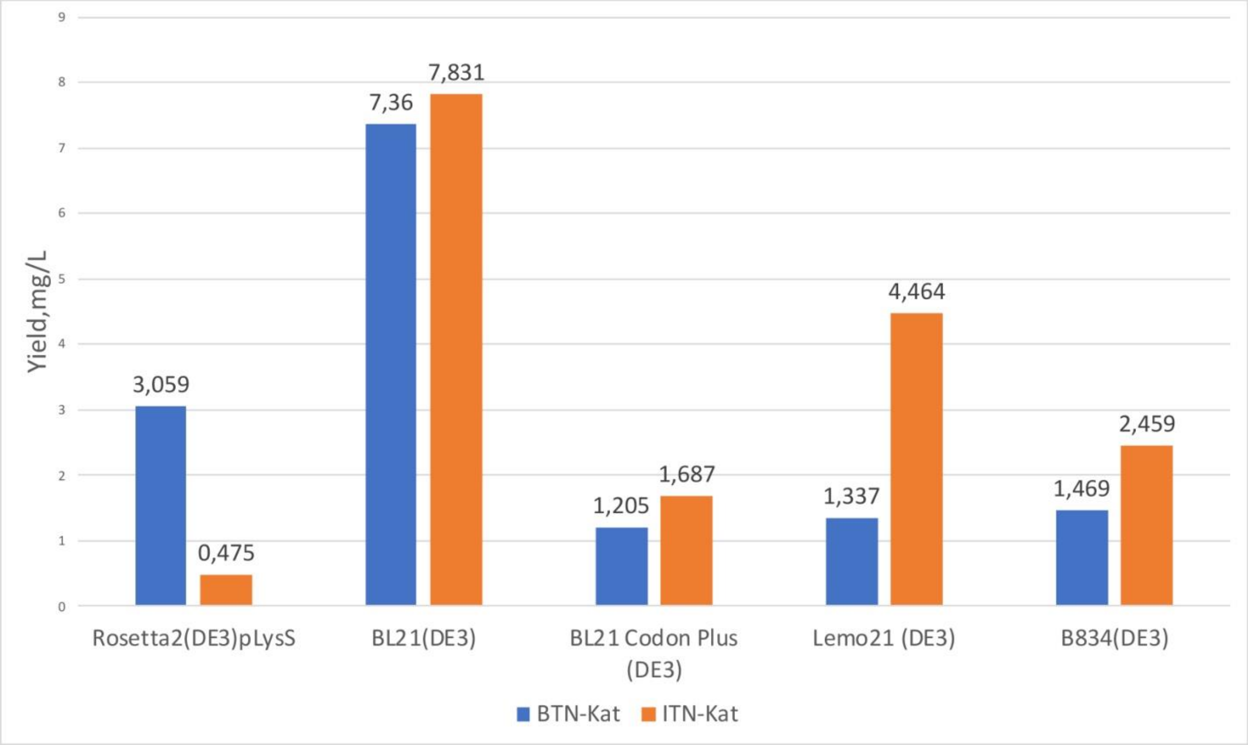

Figure S2: The yields of the BTN-Kat and ITN-Kat proteins purified from the various *E.coli* strains

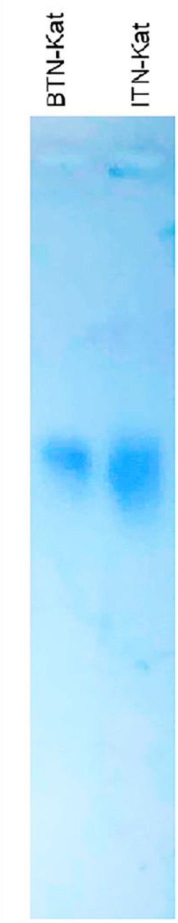

Figure S3: Native electrophoresis of BTN-Kat and ITN-Kat.

Result S2: The therapeutic effect of BTN-Kat and ITN-Kat proteins. To assess the therapeutic effect of BTN-Kat and ITN-Kat proteins, a liver histology of the mice was performed (Figure S3).

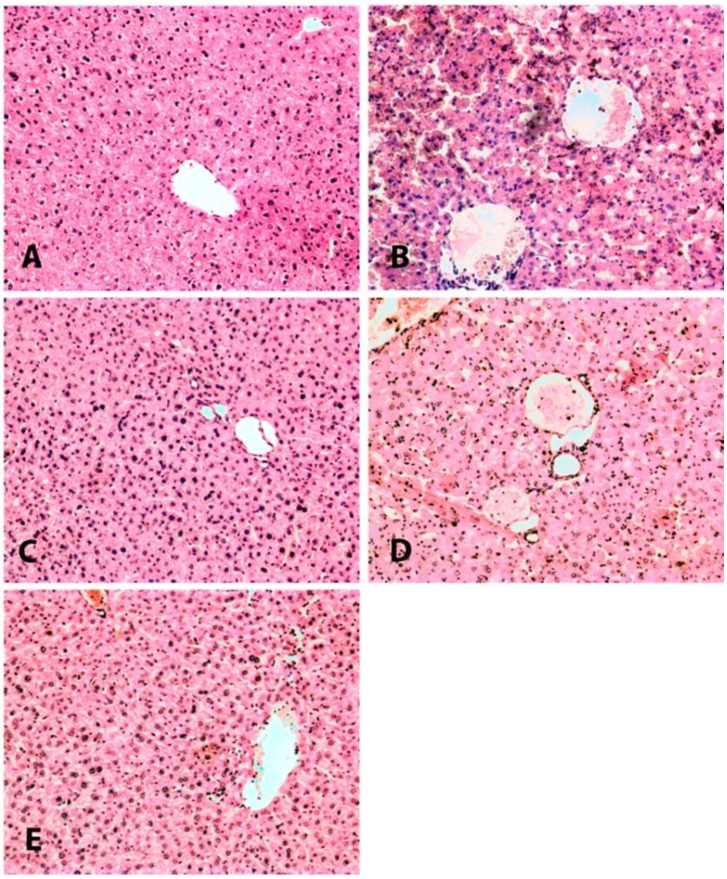

Figure S4: Liver histology (H&E stain) of mice, who received (A) i/p injection of PBS only, (B) i/p injection of LPS/D-gal, (C) i/p injection of infliximab (150 pM) followed by LPS/D-Gal injection, (D) i/p injection of BTN-Kat (150 pmol/g) followed by LPS/D-Gal injection, and (E) i/p injection of ITN-Kat (150 pmol/g) followed by LPS/D-Gal injection

Compared to normal mice liver (Figure S3A), the liver of mice injected with BTN-Kat followed by LPS/D-Gal challenge (Figure S3D) underwent destructive changes like the liver of mice after i/p injection of LPS/D-gal only (Figure S3B). The foci of necrosis were observed, the vascular ducts were violated, and clots were presented in them. Karyorexis and karyolysis were seen in hepatocytes. These facts confirm that BTN-Kat do not affect the development of the LPS/D-Gal challenge. In contrast, the livers of mice injected with ITN-Kat (Figure S3E) or infliximab (Figure S3C) followed by LPS/D-Gal challenge had vessels with a normal structure, and the structure of hepatocytes was also close to normal. This fact confirms the protective properties of the obtained proteins in the model of LPS/D-Gal-induced acute hepatotoxicity models due to their anti-TNF activity.

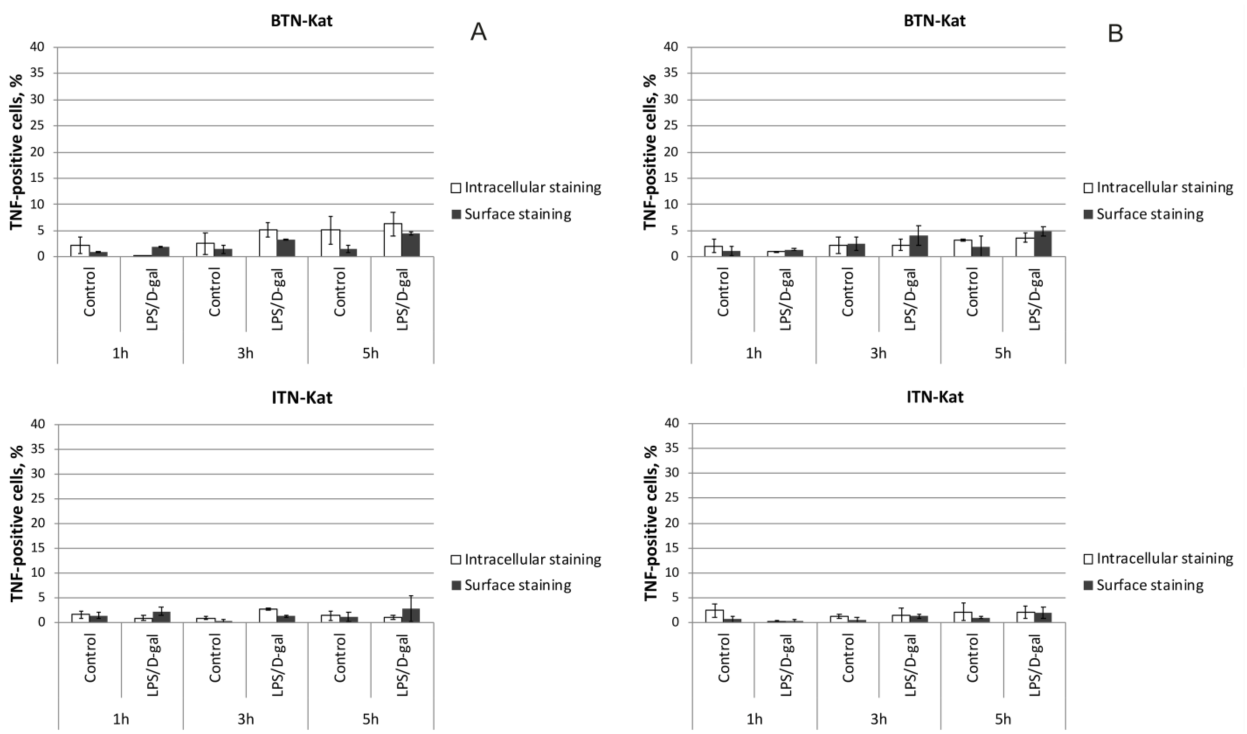

Figure S5: Levels of TNF-positive CD3(A) and CD45R-positive cells (B) 1h, 3h, and 5h after LPS/D-gal injection measured using BTN-Kat and ITN-Kat by flow cytometry surface and intracellular staining protocols. Mean levels of TNF-positive cells ±SD are plotted. Data are representative of five independent experiments.

1. Leahy, D., et al., *Structure of a fibronectin type III domain from tenascin phased by MAD analysis of the selenomethionyl protein.* Science, 1992. **258**(5084): p. 987-991.
2. Studier, F.W., et al., *Use of T7 RNA polymerase to direct expression of cloned genes.* Methods Enzymol, 1990. **185**: p. 60-89.
3. Wagner, S., et al., *Tuning Escherichia coli for membrane protein overexpression.* Proc Natl Acad Sci U S A, 2008. **105**(38): p. 14371-14376.
4. Liu, S., et al., *Removal of Endotoxin from Recombinant Protein Preparations.* Clinical Biochemistry, 1997. **30**(6): p. 455-463.
5. Putt, F.A., *Histopathologic Technic and Practical Histochemistry.* The Yale Journal of Biology and Medicine, 1954. **27**(1): p. 73-73.
